# Usability, Acceptability, and Satisfaction of a Wearable Activity Tracker in Older Adults: Observational Study in a Real-Life Context in Northern Portugal

**DOI:** 10.2196/26652

**Published:** 2022-01-26

**Authors:** Célia Domingos, Patrício Costa, Nadine Correia Santos, José Miguel Pêgo

**Affiliations:** 1 Life and Health Sciences Research Institute (ICVS) School of Medicine University of Minho Braga Portugal; 2 ICVS/3B's, PT Government Associate Laboratory Braga/Guimarães Portugal; 3 iCognitus4ALL – IT Solutions Braga Portugal; 4 Clinical Academic Center – 2CA-B Braga Portugal; 5 Associação Centro de Medicina P5 School of Medicine University of Minho Braga Portugal

**Keywords:** user experience, Technology Acceptance Model, health monitoring, fitness trackers, aging, seniors

## Abstract

**Background:**

The use of activity trackers has significantly increased over the last few years. This technology has the potential to improve the levels of physical activity and health-related behaviors in older adults. However, despite the potential benefits, the rate of adoption remains low among older adults. Therefore, understanding how technology is perceived may potentially offer insight to promote its use.

**Objective:**

This study aimed to (1) assess acceptability, usability, and user satisfaction with the Xiaomi Mi Band 2 in Portuguese community-dwelling older adults in a real-world context; (2) explore the mediating effect of the usability on the relationship between user characteristics and satisfaction; and (3) examine the moderating effect of user characteristics on the relationship between usability and user satisfaction.

**Methods:**

Older adults used the Xiaomi Mi Band 2 over 15 days. The user experience was evaluated through the Technology Acceptance Model 3, System Usability Scale, and User Satisfaction Evaluation Questionnaire. An integrated framework for usability and user satisfaction was used to explore user experience. Statistical data analysis included descriptive data analysis, reliability analysis, confirmatory factor analysis, and mediation and moderation analyses.

**Results:**

A sample of 110 older adults with an average age of 68.41 years (SD 3.11) completed the user experience questionnaires. Mean user acceptance was very high—perceived ease of use: 6.45 (SD 0.78); perceptions of external control: 6.74 (SD 0.55); computer anxiety: 6.85 (SD 0.47); and behavioral intention: 6.60 (SD 0.97). The usability was excellent with an average score of 92.70 (SD 10.73), and user satisfaction was classified as a good experience 23.30 (SD 2.40). The mediation analysis confirmed the direct positive effect of usability on satisfaction (*β*=.530; *P*<.01) and the direct negative effect of depression on usability (*β*=–.369; *P*<.01). Lastly, the indirect effect of usability on user satisfaction was higher in individuals with lower Geriatric Depression Scale levels.

**Conclusions:**

Findings demonstrate that the Xiaomi Mi Band 2 is suitable for older adults. Furthermore, the results confirmed usability as a determinant of satisfaction with the technology and extended the existing knowledge about wearable activity trackers in older adults.

## Introduction

### Background

Wearable devices are electronic devices that allow users to automatically track and monitor their physical fitness metrics, including number of steps, level of activity, walking distance, calories burned, heart rate, and sleep patterns [1-4]. Over the last few years, these devices have also become increasingly popular among researchers interested in assessing and intervening on physical activity (PA)–related behaviors in real-world contexts. Wearable devices offer the opportunity to collect objective PA data in a less intrusive and inexpensive manner and provide tailored and personalized interventions in real-time [3,5,6]. In fact, overall, academic and industry research has shown that their use can increase PA levels and promote a healthier lifestyle through real-time self-monitoring of health-related behaviors [3,5,7-10]. However, despite these potential benefits, older adults still show slow technology adoption rates [10,11], possibly because these technologies are mainly developed for a younger target group, without considering health psychology or gerontology theories [7]. Consequently, older adult users may have usability barriers to technology adoption [4,12]. Furthermore, factors associated with normal aging, such as physical and cognitive decline, could limit the ability to use the technology [11].

A better understanding of older adults’ intentions to use activity trackers, and examining actual usage behavior, is becoming increasingly relevant; however, only a few studies have been conducted to determine older adults’ perceptions [7,10,13,14]. Therefore, this study aimed to understand the user experience and acceptability of an activity tracker (Xiaomi Mi Band 2), throughout daily life activities, in a cohort of community-dwelling older adults.

### Theoretical Framework

After carrying out a literature search, 3 major key concepts were identified regarding user experience and technology adoption: technology acceptance, usability, and user satisfaction. Variables regarding user characteristics were also selected, such as cognitive function, mood, and education, which may significantly influence user experience to develop our model. Thus, the theoretical framework was designed to explore older adults’ user experience with the Xiaomi Mi Band 2, by combining different theories as next described, while also enabling the examination of the impact of usability and individual characteristics on user satisfaction with the technology.

### Technology Acceptance Model

Technology acceptance is an important factor in determining the long-term adoption of activity trackers [3]. The Technology Acceptance Model (TAM) is the most applied theoretical model for evaluating or predicting users’ acceptance of new technologies. The TAM was adapted from the Theory of Reasoned Action [15] and was initially developed by Davis [16]. This model assumes that the perceived ease of use (PEOU) and perceived usefulness (PU) are the primary factors influencing an individual’s intention to use new technology [3,12,16]. PEOU refers to the degree to which a person perceives how easy it is to use the technology, and PU refers to how using the technology will improve performance [16]. Moreover, PEOU and PU can be influenced by various external factors, including both the device and user characteristics [3,16,17]. The usability seems to be predictive of acceptance regarding the device characteristics because they directly relate to the PEOU and PU and may moderate attitudes and behavioral intentions (BIs) to use a system [3].

The original TAM was extended to TAM 2 by Venkatesh and Davis [18] to explain PU and usage intentions in terms of social influence and cognitive instrumental determinants. Later, Venkatesh and Bala [19] updated the model, including other variables affecting PEOU, such as individual differences (computer self-efficacy, computer anxiety [CANX], and computer playfulness), perceptions of external control (PEC), and system characteristics–related adjustments (perceived enjoyment and objective usability).

### System Usability Scale

Initially proposed by John Brooke in 1986, the System Usability Scale (SUS) is the most widely used standardized questionnaire to measure perceived usability [8,17,20,21]. Recent literature shows that several studies extend the TAM by incorporating the SUS [17,22,23]. Although the SUS has been assumed to be unidimensional, recent research reveals that the SUS has 2 subscales—usability and learnability—with items 4 and 10 providing the learnability dimension and the other 8 items the usability dimension [24,25].

According to ISO-9241-11 [26], usability refers to the effectiveness, efficiency, and user satisfaction rating of a product in a specific environment by a particular user for a particular purpose. More precisely, effectiveness refers to which of the system’s intended goals can be achieved; efficiency is the effort required for a user to achieve the goals; and satisfaction depends on how comfortable the user feels using the system [8,21,27]. Therefore, usability is a critical factor that directly affects the use and adoption of technology by older adults.

### User Satisfaction Evaluation Questionnaire

The literature on technology acceptance has included many model variants and extensions, including user satisfaction as a key indicator of user acceptance [28-34]. Moreover, satisfaction has been described as a predictor of behavior intention [29]. The User Satisfaction Evaluation Questionnaire (USEQ) was initially designed by Gil-Gómez et al [35] to evaluate the satisfaction of the users with virtual rehabilitation systems. Recently, the USEQ was adapted and validated into European Portuguese by Domingos et al [36] to evaluate an activity tracker (Xiaomi Mi Band 2) in older adults, showing psychometric properties consistent with the original version.

### User Characteristics

In a theoretical framework developed by Venkatesh and Bala [19], individual differences, such as personality and demographics (eg, traits or individuals’ states, gender, and age), were suggested to inﬂuence individuals’ perceptions of PU and PEOU. Specifically, personality is related to individual differences in cognitive, emotional, and motivational aspects of mental states that result in stable behavioral action [37]. Moreover, personality has been found to affect technology perceptions and acceptance [3,38].

Additionally, older individuals may show age-related declines, including attention, memory, and processing speed, which may further impact how they interact with the technology [3]. The aging process is also associated with a decline in visual faculties, that is, visuospatial functioning, visual acuity, color discrimination, and contrast sensitivity, crucial for learning new information and executing technology-based tasks [39]. Thus, researchers have focused on the impact of cognitive abilities, self-efficacy, and technology-related anxiety in technology acceptance [11]. Lastly, compared with younger adults, the senior population may be more resistant to adopt new technologies due to cultural factors, education, and experience [3].

### Research Framework and Hypotheses

**Figure 1:**
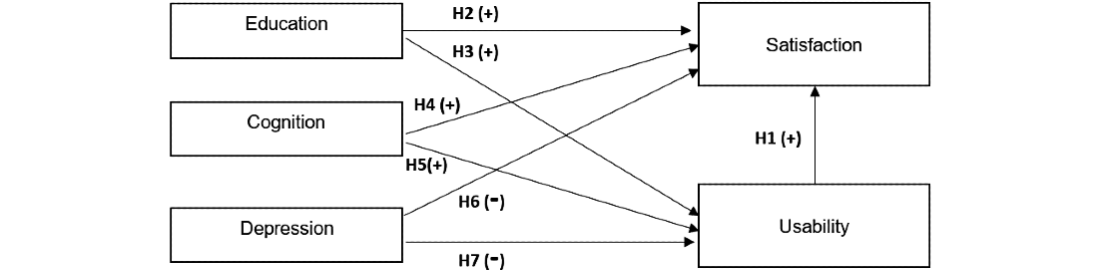
Research hypothesis framework.

This study uses a model based on the SUS to measure usability and the USEQ to measure user satisfaction and incorporate individual characteristics, such as education, mood, and cognitive performance (Figure 1). The design was founded on the basic theory studied to provide a clear causal relationship between the independent variables (exogenous) and the dependent variables (endogenous). The model has 5 variables exploring the user experience with the Xiaomi Mi Band 2 in older adults.

The following hypotheses were formulated:

H1: Usability has a positive effect on satisfaction.

H2: Education has a positive effect on satisfaction.

H3: Education has a positive effect on usability.

H4: Cognition has a positive effect on satisfaction.

H5: Cognition has a positive effect on usability.

H6: Depression has a negative effect on satisfaction.

H7: Depression has a negative effect on usability.

**Figure 2:**
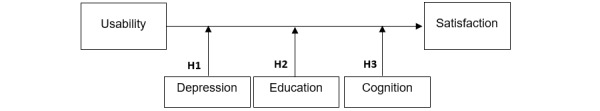
Moderating effect of user characteristics.

Additionally, user characteristics’ potential moderating effect on the direct effect between usability and satisfaction was tested separately for each variable (Figure 2).

## Methods

### Participants and Research Ethics

A priori sample size calculation and power analysis were performed using G*Power version 3.1.9.3 (Heinrich-Heine-Universität Düsseldorf). Considering that the study is part of a larger project, which used a wearable device to measure and quantify free-living PA in older adults, a total of 120 participants were determined assuming an effect size of 0.32 [40-42], an *α* of .05, power of 0.95, and dropout rate of 23%. The power analysis for this user experience study was conducted considering the sample size calculated previously and a medium effect size [23] confirmed a power of 0.92. Moreover, the rule of thumb to determine sample size in multiple regression analyses confirmed that the minimum sampling requirements for the analysis were met [43]. Therefore, a total of 120 participants, representative of the general older Portuguese population living in the community within the age group 65-74 years, were recruited from health centers and local gyms in Northern Portugal. The older adults were defined according to the World Health Organization, which considers older people, in developed economies, as those aged 65 or older. To reduce variability due to the age effect, we used the first 10-year age group in the same way as Eurostat publication—Ageing Europe [44].

The applied exclusion criteria comprised inability to understand informed consent; diagnosed neuropsychiatric and neurodegenerative disorders; or disability that limited independent walking, visual, auditory, or fine motor skills. Participants having previous experience with other wearable activity trackers were not excluded from the study. A final sample of 110 participants was enrolled in the study. The study ran from April 2018 to July 2019.

The study was conducted according to the Helsinki Declaration and approved by the local and ethics committees (Approval Number 42-2018), developed in compliance with the new General Data Protection Regulation, and approved by the Portuguese Data Protection Authority (Approval Number 11286/2017). Study goals and assessments were explained during screening procedures. All participants provided written informed consent before study enrollment, which included consent to their data processing.

### Data Collection and Instruments

A baseline characterization was performed through a sociodemographic questionnaire, and a neuropsychological evaluation to obtain mood (Geriatric Depression Scale [GDS]) [45] and global cognitive profiles (Mini-Mental State Examination [MMSE]) [46]. For screening “cognitive impairment” via the MMSE, the following cutoff values were used: individuals with no education, <15 points; 1-11 years of school completed, <22 points; and >11 years of school completed, <27 points [47]. For assessment of the presence of depressive symptomatology via the GDS, the cutoff value considered was a total number of depressive symptoms over 11 [48].

To assess the users’ experience, the Xiaomi Mi Band 2 was provided to participants that should be worn continuously for over 15 days, while performing their normal daily activities. The wearable was returned after the testing period for data analysis. In other studies, testing periods range from 3 to 7 days [13,49-51]; because a 7-day testing period corresponds to a short-term user experience, we decided to extend this period to 15 days. Upon completing the usage period, participants were also asked to provide information about their user experience. The TAM 3 [19] was used to collect information about technology acceptance, the SUS [25] for perceived usability, and the USEQ [35,36] for user satisfaction.

### Xiaomi Mi Band 2

The selection of wearable activity tracker was based on a review of several different commercially available devices on the market [8,52,53]. The selection criteria included their popularity in the health tracking device market, availability, continuous monitoring of PA without a smartphone, price, battery life, various data captured via sensors, and ability to export data. The Xiaomi Mi Band 2 was selected because, at the study time, it offered the best price-quality ratio, had an estimated battery life of almost 30 days, was ergonomic, accessible, easy to operate, and did not require continuous communication with a smartphone. The system combines sensors that allow the objective assessment of daily free-living PA, with its algorithms calculating steps, intensity, energy expenditure, and distance traveled [49,53,54].

### Technology Acceptance Model

The TAM 3 was adapted to the context of the use of activity tracking technologies by older adults, and the key dimensions of acceptance were investigated using the following constructs: PEOU, PEC, CANX, BI, and USE. PEOU was measured using all 4 items adapted from the TAM 3; PEC using 2; CANX using 3, BI and USE were measured using the only item on the original scale. Multimedia Appendix 1 presents a list of items for all the constructs. TAM items were measured on a 7-point Likert scale, starting from “1=strongly disagree” to “7=strongly agree”. The mean scores of each item were computed and the mean of means of each construct was calculated and used to perform statistical analysis [19].

### System Usability Scale

The SUS is a 10-item questionnaire, consisting of 5 positive and 5 negative statements, with the 5 responses for each statement ranging from “5=strongly agree” to “1=strongly disagree” (Multimedia Appendix 2). The SUS score is calculated by taking 1 from all the scores on odd-numbered items and subtracting 5 from the even-numbered items scores. The sum of the scores is then multiplied by 2.5 to give an overall SUS score, and range from 0 (extremely poor usability) to 100 (excellent usability) [21,25]. The value of 68 is considered the average for the SUS score; a score above or less than 68 is considered above average or below average, respectively [55,56]. The grade rankings of scores proposed by Bangor et al [56] were here used to provide a more meaningful basis for the SUS score interpretation.

### User Satisfaction Evaluation Questionnaire

The USEQ is a 6-item questionnaire with a 5-point Likert Scale (Multimedia Appendix 3). The total score ranges from 6 (poor satisfaction) to 30 (excellent satisfaction). All items are affirmative, except item 5, which is a negative item. The numerical value of the affirmative items is used to calculate the score. The negative item subtracts the numerical value of the response from 6 and then adds this result to the total score. The USEQ score is evaluated using the following classification: poor (0-5), fair (5-10), good (10-15), very good (15-20), or excellent (20-25) satisfaction [35,36].

### Statistical Analysis

#### Overview

The statistical analysis was organized to address the following aims: (1) explore the mediating effect of the usability on the relationship between user characteristics and satisfaction; and (2) examine the moderating effect of user characteristics on the relationship between usability and user satisfaction. Briefly, the statistical analysis was performed according to the following steps: (1) descriptive statistics; (2) instruments’ psychometric proprieties; (3) structural equation modeling (SEM); and (4) moderation analysis.

#### Descriptive Statistics

Descriptive data analysis was performed using IBM SPSS Statistics (version 26) to depict the characteristics of the study. Descriptive statistics, including frequency, percentage, mean, standard deviation, minimum, maximum, skewness, and kurtosis, were calculated for each variable. Normality was considered adequate if absolute values for skewness and kurtosis were above 3.0 and 10.0, respectively [57,58]. The percentage of missing values across the variables was analyzed. Methods for handling missing data were not applied because there were no missing data.

#### Instruments’ Validation

Before structural modeling, the measurement model of latent variables for their dimensionality/structure and reliability was assessed.

Confirmatory factor analysis (CFA) was conducted using JASP (version 0.11.1; JASP Team, University of Amsterdam) to examine the structure of the SUS (used to measure usability) and USEQ (used to measure user satisfaction). Variables with factor loadings above 0.4 were included. To assess the goodness of fit of the model, the following indices and thresholds were applied: chi-square (*χ*^2^, *P*>.05), *χ*^2^/degrees of freedom (*df*) ratio (≤3), Comparative Fit Index (CFI ≥0.90), Tucker–Lewis Index (TLI ≥0.90), Goodness-of-Fit Index (GFI ≥0.90), root mean squared error of approximation (RMSEA <0.08), and standardized root mean squared residual (SRMR ≤0.08) [59-61].

Reliability analysis was performed using IBM SPSS Statistics (version 26) to analyze the internal consistency of item responses of the SUS and USEQ instruments. Reliability was estimated using the McDonald omega (*ω_t_*) coefficient [62,63]. Given ordinal response format items, the McDonald omega coefficient (*ω_t_*) provides more accurate estimates of reliability than Cronbach *α* [62,64,65]. Coefficients values over 0.70 are considered indicators of satisfactory item homogeneity [65,66].

#### Structural Equation Modeling

SEM was applied to check the hypothesis relationship between the proposed factors that directly and indirectly influence older adult’s user satisfaction (structural model) with technology. SEM allows to analyze the structural relationship between measured variables and latent variables. The derived scores for usability and user satisfaction were supported by CFA.

Data were analyzed using IBM SPSS AMOS (version 25) and the parameters were estimated by the maximum likelihood method. The significance level of 5% was used as a threshold for the research proposition testing. To determine whether the model was reasonable and acceptable, the following indices were considered: *χ*^2^, *χ^2^/df* ratio, CFI, TLI, GFI, and RMSEA. The criteria for an acceptable model fit were the same as those reported for the CFA.

To assess multicollinearity, the inspection of the correlation matrix of the predictor variables (education, MMSE, GDS, and usability) and the analysis of the variance inflation factor (VIF) and tolerance were performed (IBM SPSS Statistics, version 26). The tolerance values close to 1 were considered as an indicator of low multicollinearity, whereas a value close to 0 as a potential indicator of collinearity problem [67,68]. Moreover, VIF=1 was considered an indicator that the independent variables are not correlated, and 1<VIF<5 an indicator that the variables are moderately correlated with each other [67].

#### Moderation Analysis

Moderation analysis was performed to examine whether the relationship between usability (predictor) and user satisfaction (outcome variable) depended on user characteristics (moderator). The analysis was performed using the MedMod package in jamovi (version 1.2.27; The jamovi Project) software. The significance of the interaction term of usability on user satisfaction at specific values (–1 SD, mean, +1 SD) of GDS, education, and MMSE (moderators) was assessed, exploring when the effect of usability on user satisfaction depends on the level of the moderating test variable.

## Results

### Study Participants

A total of 110 participants completed the final assessment after the testing period. Table 1 summarizes the demographic, mood, and global cognitive characteristics of the sample. Participants had a mean age of 68.41 (SD 3.11) years, and 45.5% (50/110) were identified as males. The mean years of formal education were 7.95 (SD 5.38).

**Table 1:** Characteristics of the study participants (N=110).

Characteristics		Values
**Gender**		
	Male, n (%)	50 (45.5)
**Age (years), mean (SD)**		68.41 (3.11)
	64-70, n (%)	73 (66.4)
	≥70, n (%)	37 (33.6)
**Education (years), mean (SD)**		7.95 (5.38)
	1-4, n (%)	58 (52.7)
	5-11, n (%)	24 (21.8)
	≥12, n (%)	28 (25.5)
**MMSE^a^ (total score), mean (SD)**		26.95 (2.00)
	22-27, n (%)	41 (37.3)
	≥27, n (%)	69 (62.7)
**GDS^b^ (total score), mean (SD)**		6.05 (4.58)
	>11, n (%)	17 (15.5)

^a^MMSE: Mini-Mental State Examination.

^b^GDS: Geriatric Depression Scale.

### Instruments’ Descriptive Statistics

The results of descriptive statistics for the instruments (TAM 3, SUS, and USEQ) are presented in Tables 2-4, respectively. The skewness and kurtosis values indicate some degree of non-normality. In reality, most behavioral research data do not follow univariate normal distributions [69,70]. Moreover, the results reveal a severe violation of normality for the following items and constructs: USEQ 1, SUS 1, SUS 3, SUS 5, SUS 9, PEOU 3, PEC, and CANX. Thus, these were excluded from further path analysis.

**Table 2:** Descriptive statistics for Technology Acceptance Model 3 items.

Items			Range		Mean (SD)			Skewness				Kurtosis			
**Perceived Ease of Use (PEOU)**															
	PEOU 1	2-7		6.28 (1.08)			–1.62				2.25				
	PEOU 2	1-7		6.06 (2.01)			–1.97				2.22				
	PEOU 3	3-7		6.84 (0.60)			–4.37				20.92				
	PEOU 4	3-7		6.60 (0.92)			–2.42				5.11				
	PEOU score	3.50-7.00		6.45 (0.78)			–1.48				1.54				
	PEOU final	3.67-7.00		6.31 (0.94)			–1.25				0.24				
**Perceptions of External Control (PEC)**															
	PEC 1	3-7		6.55 (0.97)			–2.38				5.19				
	PEC 2	4-7		6.94 (0.41)			–6.84				46.91				
	PEC score	4.00-7.00		6.74 (0.55)			–2.62				7.34				
**Computer Anxiety (CANX)**															
	CANX 1	6-7		6.99 (0.10)			–10.49				110.00				
	CANX 2	1-7		6.86 (0.83)			–6.72				45.64				
	CANX 3	2-7		6.71 (0.97)			–3.49				11.44				
	CANX score	4.33-7.00		6.85 (0.47)			–3.55				12.72				
**Behavioral intention (BI)**															
	BI	1-7		6.60 (0.97)			–3.00				10.84				
	USE (hours)	13-24		23.85 (1.12)			–8.99				85.16				

**Table 3:** Descriptive statistics for System Usability Scale items.

Items	Range	Mean (SD)	Skewness	Kurtosis
1	1-5	4.71 (0.65)	–2.82	9.87
2	1-5	1.44 (1.03)	2.40	4.70
3	2-5	4.92 (0.36)	–5.81	40.38
4	1-5	1.38 (1.04)	2.51	4.75
5	1-5	4.85 (0.56)	–4.47	23.01
6	1-5	1.28 (0.83)	3.12	9.26
7	1-5	3.58 (0.78)	–2.15	4.88
8	1-5	1.30 (0.92)	3.06	8.16
9	3-5	4.86 (0.46)	–3.42	10.71
10	1-5	1.41 (1.08)	2.52	4.95
System Usability Scale score	55-100	92.70 (10.73)	–1.61	1.77

**Table 4:** Descriptive statistics for User Satisfaction Evaluation Questionnaire items.

Items	Range	Mean (SD)	Skewness	Kurtosis
2	3-5	4.82 (0.47)	–2.66	6.46
3	2-5	4.65 (0.71)	–2.21	4.50
4	2-5	4.47 (0.75)	–1.30	0.98
5	1-5	4.65 (0.93)	–2.71	6.15
6	1-5	1.28 (0.83)	3.12	9.26
User Satisfaction Evaluation Questionnaire score	14-25	23.30 (2.40)	–1.81	2.99

### Instruments’ Psychometric Proprieties

As reported by Domingos et al [36], the CFA supported the conceptual unidimensionality of the USEQ (*χ*^2^_4_=1.83, *P*=.12, *χ*^2^/*df*=1.83; CFI=0.973, TLI=0.931, GFI=0.977, RMSEA=0.087, SRMR=0.038). Furthermore, the CFA for the SUS showed satisfactory values for the following indexes: CFI=0.816, GFI=0.928, and SRMR=0.074. The fit indices for the model are presented in Table 5.

Regarding internal consistency, for the SUS questionnaire reliability was calculated only with items included in path analysis (SUS 2, SUS 4, SUS 6, SUS 7, SUS 8, SUS 10). Moreover, the USEQ showed acceptable reliability (Cronbach *α*=.677; McDonald *ω*=0.722), as reported by Domingos et al [36]. The McDonald *ω* coefficients showed acceptable values for the SUS and USEQ questionnaires ranging from 0.712 to 0.722, respectively.

**Table 5:** Confirmatory factor analysis for instruments.

Fit indices	User Satisfaction Evaluation Questionnaire	System Usability Scale
*χ* ^2^	7.313	30.074
*df*	4	9
*χ^2^/df*	1.83	3.34
*P* value	.120	<.001
Comparative Fit Index	0.973	0.816
Tucker–Lewis Index	0.931	0.694
Goodness-of-Fit Index	0.977	0.928
Root mean squared error of approximation	0.087	0.146
Standardized root mean squared residual	0.038	0.074

### Users’ Experience

The high ratings of the TAM 3 indicate excellent technology acceptance by the participants. Overall, the average ratings for user experience with the Xiaomi Mi Band 2 were 6.45 (SD 0.78) for PEOU, 6.74 (SD 0.55) for PEC, 6.85 (SD 0.47) for CANX, and 6.60 (SD 0.97) for BI. Furthermore, the participants reported an average of 23.85 (SD 1.12) hours of use per day (Table 2). These results indicate that participants found that the Xiaomi Mi Band 2 is an easy-to-use and easy-to-control device, potentially perceiving its usefulness regarding health benefits and having the intention to use it in the future.

Regarding usability, the overall SUS score ranged from 55 to 100 (mean [SD] 92.70 [10.73]), with 96% (106/110) of the participants reporting a score above the acceptability baseline of the SUS. Moreover, 45.5% (50/110) of the participants classified the activity tracker achieved as best imaginable (Table 6). Thus, these results suggest that the Xiaomi Mi Band 2 is a usable wearable activity tracker among older adults.

Finally, all participants reported a user satisfaction experience above the USEQ baseline value defined as a good experience, with a mean USEQ score of 23.30 (SD 2.40; Table 4). Moreover, 85.5% (94/110) of the participants rated the satisfaction with the Xiaomi Mi Band 2 as excellent (Table 6). Still, despite older adults reporting good satisfaction with the device, concerns were noted regarding the clarity of the technology’s information.

**Table 6:** User experience classification for usability and satisfaction (N=110).

Classification		Value, n (%)
**Usability (System Usability Scale)**		
	Ok	8 (7.3)
	Good	16 (14.5)
	Excellent	36 (32.7)
	Best imaginable	50 (45.5)
**Satisfaction (User Satisfaction Evaluation Questionnaire)**		
	Good	2 (1.8)
	Very good	14 (12.7)
	Excellent	94 (85.5)

### The Structural Equation Modeling for User Satisfaction

Table 7 shows fit indexes for the structural model, showing acceptable values for the *χ^2^/df* (1.67) and RMSEA (0.079) indexes and values slightly less than the threshold for a good model fit for the following indexes: GFI=0.880, TLI=0.818, and CFI=0.868. Based on these indexes, the model has a moderate acceptable fit.

**Figure 3:**
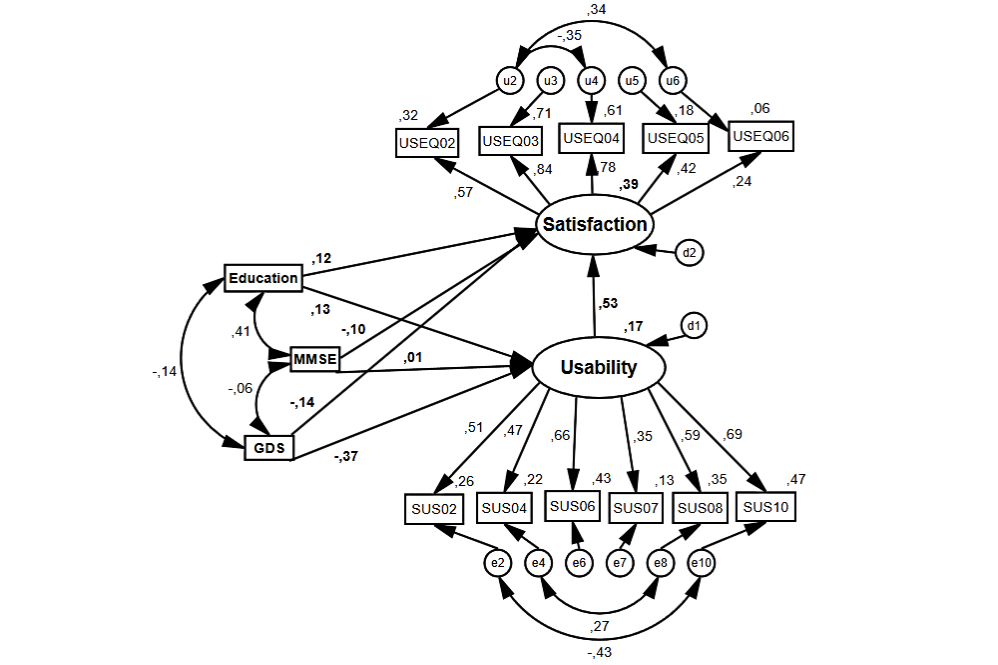
Path diagram for the research model. GDS: Geriatric Depression Scale; MMSE: Mini-Mental State Examination; SUS: System Usability Scale; USEQ: User Satisfaction Evaluation Questionnaire.

The path diagram of the model is presented in Figure 3. Coefficients within paths are standardized coefficients from regressions. Table 8 summarizes the results of hypothesis testing, including standardized coefﬁcients and significance levels. Specifically, results show that usability was significantly and positively associated with user satisfaction (*β*=.530; *P*<.01), thereby supporting Hypotheses 1. By contrast, depression was significantly and negatively associated with usability (*β*=–.369; *P*<.01), supporting Hypotheses 7.

**Table 7:** Fit indices for the hypothesized model.

Model fit index	Value
*χ* ^2^	110.475
*df*	66
*χ^2^/df*	1.67
*P* value	<.001
Goodness-of-Fit Index	0.880
Tucker–Lewis Index	0.818
Comparative Fit Index	0.868
Root mean squared error of approximation	0.079

Individual characteristics (education, cognition, and depression) collectively explained 16.8% of usability variance. Furthermore, individual characteristics and usability collectively explained 39.1% of the variance in satisfaction. Specifically, depression negatively impacted usability and satisfaction, with a significant effect on usability (*β*=–.369; *P*<.01); while, regarding education, a positive, but not significant, usability and satisfaction effect was observed (education > satisfaction: *β*=–.121; *P*<.23; education > usability: *β*=–.130; *P*<.25). Despite confirming the theoretical model, most research hypotheses were not statistically proven with adequate goodness of fit. Nonetheless, usability seems to be a strong predictor of user satisfaction.

Considering the possible multicollinearity issues in the SEM, the absolute values of correlation coefficients were calculated and ranged from 0.009 to 0.45. The tolerance values ranged from 0.82 to 0.87 and the VIF values from 1.15 to 1.22, indicating that no independent variable is in a perfect linear function with other any independent variable.

**Table 8:** Results of hypothesis testing based on standardized path coefficients for the research model.

Hypothesis	Estimate	Standard error	Critical ratio	*P* value
H1: Usability > Satisfaction	0.530	0.089	3.008	.003
H2: Education > Satisfaction	0.121	0.005	1.194	.23
H3: Education > Usability	0.130	0.011	1.147	.25
H4: Cognition > Satisfaction	–0.098	0.013	–0.999	.32
H5: Cognition > Usability	0.011	0.029	0.104	.92
H6: Depression > Satisfaction	–0.140	0.006	–1.376	.17
H7: Depression > Usability	–0.369	0.014	–3.010	.003

### Moderation Analysis

#### Moderating Effect of the GDS

**Figure 4:**
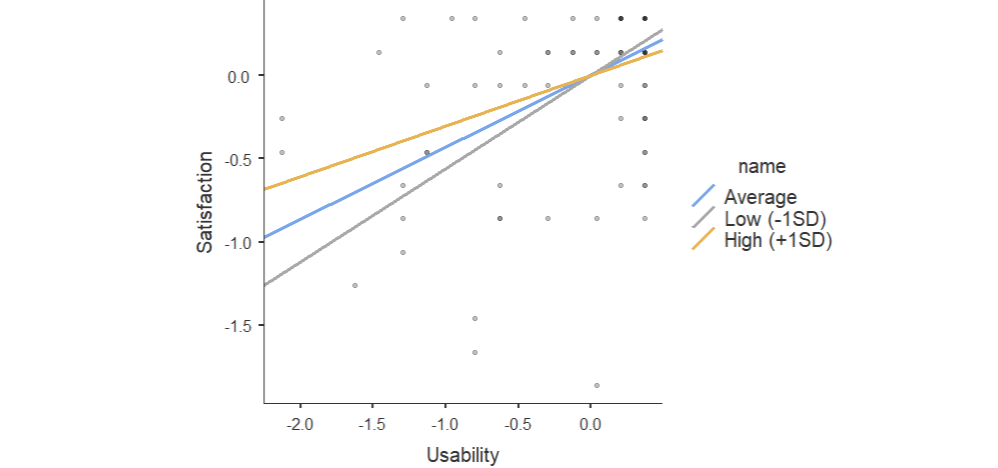
Simple slope plot for the moderating effect of Geriatric Depression Scale (GDS) and usability in the prediction of user satisfaction.

Usability significantly predicted satisfaction (*β*=.43; *P*<.001; Table 9). The interaction effect of usability × GDS was not significant (*β*=–.028; *P*<.06); however, because the *P*-value is approximately .05, we can conclude there is a tendency to infer that the effect of the satisfaction is dependent on GDS levels. The simple slopes of the interaction at –1 SD, mean, and +1 SD of GDS are plotted in Figure 4. Results indicate a signiﬁcant association for high and low values of low GDS, respectively, in the same direction (*β*=.30, *P*<.001; *β*=.56, *P*<.001; Table 10). Moreover, the effect of usability on user satisfaction through the GDS was higher in individuals with lower GDS levels.

**Table 9:** Estimates for the moderating effect of the GDS^a^ and usability in the prediction of user satisfaction.

Variable	Estimate	Standard error	Z	*P* value
Usability	0.43	0.082	5.28	<.001
GDS	–0.012	0.009	–1.34	.18
H1: Usability × GDS	–0.028	0.015	–1.90	.06

^a^GDS: Geriatric Depression Scale.

**Table 10:** Effect of the usability on satisfaction at different levels of the GDS^a^.

Effect	Estimate	Standard error	Z	*P* value
Average	0.43	0.083	5.22	<.001
Low (–1 SD)	0.56	0.135	4.16	<.001
High (+1 SD)	0.30	0.070	4.36	<.001

^a^GDS: Geriatric Depression Scale.

#### Moderating Effect of Education

**Figure 5:**
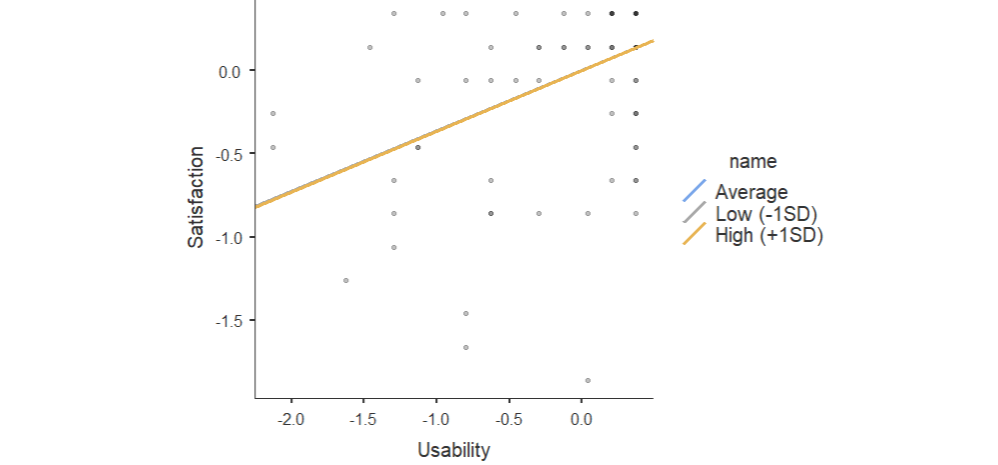
Simple slope plot for the moderating effect of education and usability in the prediction of user satisfaction.

Usability significantly predicted satisfaction (*β*=.36; *P*<.001; Table 11). However, the interaction effect of usability × education in the direct path between usability and user satisfaction was not significant (*β*=3.63 × 10^–4^; *P*<.98). Results from simple slope estimates for the effect of usability on satisfaction indicated that education did not moderate the relationship between these variables (Table 12). Moreover, the interaction plot (Figure 5) showed no difference in simple slopes at –1 SD, mean, and +1 SD.

**Table 11:** Estimates for the moderating effect of education and usability in the prediction of user satisfaction.

Variable	Estimate	Standard error	Z	*P* value
Usability	0.36	0.070	5.23	<.001
Education	0.004	0.008	0.522	.60
H2: Usability × Education	3.63 × 10^–4^	0.017	0.022	.98

**Table 12:** Effect of the usability on satisfaction at different levels of education.

Effect	Estimate	Standard error	Z	*P* value
Average	0.364	0.067	5.23	<.001
Low (–1 SD)	0.362	0.098	3.71	<.001
High (+1 SD)	0.366	0.128	2.85	.004

#### Moderating Effect of the MMSE

**Figure 6:**
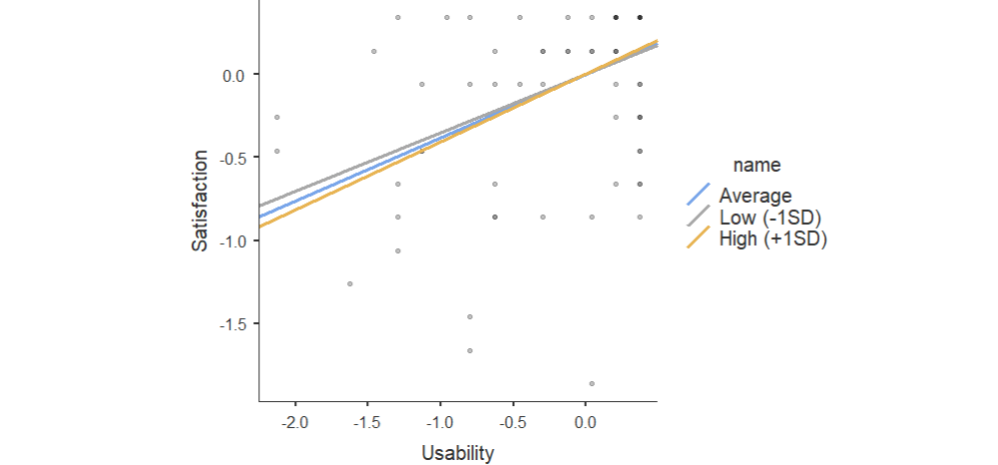
Simple slope plot for moderating effect of Mini-Mental State Examination (MMSE) and usability in the prediction of user satisfaction.

The interaction effect of usability × MMSE in the direct path between usability and user satisfaction was not significant (*β*=.014; *P*<.66; Table 13). The simple slopes of the interaction at –1 SD, mean, and +1 SD of the GDS are plotted in Figure 6. Results indicate a positive relationship between usability and satisfaction for both low (*β*=.352; *P*<.001) and high (*β*=.407; *P*<.001) MMSE levels (Table 14). Additionally, the results suggested that the indirect effect of usability on user satisfaction through the MMSE is higher for individuals with higher levels of MMSE.

**Table 13:** Estimates for the moderating effect of the MMSE^a^ and usability in the prediction of user satisfaction.

Variable	Estimate	Standard error	Z	*P* value
Usability	0.380	0.070	5.46	<.001
MMSE	–0.008	0.020	–0.387	.70
H3: Usability × MMSE	0.014	0.031	0.445	.66

^a^MMSE: Mini-Mental State Examination.

**Table 14:** Effect of the usability on satisfaction at different levels of Mini-Mental State Examination (MMSE).

Effect	Estimate	Standard error	Z	*P* value
Average	0.380	0.070	5.45	<.001
Low (–1 SD)	0.352	0.079	4.44	<.001
High (+1 SD)	0.407	0.105	3.87	<.001

## Discussion

### Principal Findings

In recent years, wearable activity trackers are part of a rapidly growing trend in biomedical research and medicine [13,71,72]. These devices have been employed in behavior change interventions due to their potential to motivate individuals to comply with a daily activity goal [13,71]. Because most older adults have insuﬃcient levels of PA, these technologies may be especially beneficial in middle-aged and older age groups. Nonetheless, it is reported that only 16% of activity tracker owners are 55-64 years of age and 7% over 65 [4], indicating that possibly these devices may not be feasible or acceptable to older adults [73]. Therefore, it is necessary to understand how older adults perceive these new technologies. Thus, to better understand potential barriers to using these technologies, the acceptability, usability, and user satisfaction experience with the Xiaomi Mi Band 2 were examined in a population of older adults.

Results from users’ experience indicate an excellent technology acceptance with high ratings of the TAM 3 in all constructs, including PEOU, PEC, CANX, and BI. Previously, Puri et al [49] found a moderate level of acceptance (65%) for the Xiaomi Mi Band 2 among the Canadian community-dwelling older adults, where, interestingly, participants reported a significantly higher acceptance rate for the Xiaomi Mi Band 2 when compared with Microsoft Band. In our study, no other devices were tested, and thus, did not allow for any comparison between wearable devices.

Concerning usability, all participants scored their experience above the acceptability baseline for the SUS. Thus, these results indicate that the Xiaomi Mi Band 2 has excellent usability for older adults in this specific context. Participants also reported a user satisfaction experience above the USEQ baseline value defined as a good experience, suggesting excellent user satisfaction. Nonetheless, such a large score on the SUS, mean 92.70 (SD 10.73), was surprising. In the study by Liang et al [8], the Xiaomi Mi Band 2 was one of the devices that achieved the highest score among several selected wearable devices with distinct market performance, but its mean SUS score was 65.12 (SD 14.73). Possible explanations range from the intrinsic motivation to use the device and how the device is supplied; therefore, such aspects should be evaluated in future studies.

This study also examined factors influencing user satisfaction with the Xiaomi Mi Band 2, based on the proposed theoretical framework. The hypothetical model was supported by moderate acceptable fit indices values (*χ^2^/df*=1.67, GFI=0.880, TLI=0.818, CFI=0.868, and RMSEA=0.079). Furthermore, 2 of the testing hypotheses were proven. Overall, results indicate that usability is a significant predictor of user satisfaction (*β*=.530; *P*<.01), which, in turn, was negatively affected by depression symptoms (*β*=–.369; *P*<.01). The model shows that individual characteristics explain 16.8% of the usability variance and 39.1% of the variance in satisfaction collectively with usability. Specifically, a significant negative effect of depression on usability was found (*β*=–.369; *P*<.01).

Additionally, user characteristics’ potential moderating effect on the interaction between usability and user satisfaction was examined. Results suggested that the GDS moderates the usability effect on user satisfaction, and the effect is higher in individuals with lower GDS levels. However, we did not observe significant moderating effects for education (*β*=3.63 × 10^–4^; *P*<.98) and MMSE (*β*=.014; *P*<.66) on the interaction between usability and user satisfaction, contrary to our expectations. Future research should explore additional moderating effects through the user characteristics, including personal traits as well as motivational and cultural aspects to enable a better understanding of the factors that may influence user satisfaction and consequently facilitate technology adoption.

Overall, our results align with a recent study investigating the impact of depressive symptoms on web user experience measures, indicating that mood may be a factor influencing technology usability [74]. Additionally, recent research investigating the relationship between user perceptions and user characteristics has shown that older adults demonstrate positive attitudes toward mobile technologies and report technologies’ complexity. User characteristics, such as age, processing speed, and attention, significantly influence older adults’ usage behavior. Furthermore, the education level was found to be positively correlated with the diversity of use. Probably, individuals with higher education levels are typically more motivated to accept new concepts. The authors also mentioned that the usability problems could be attributed to poor memory, decreased vision, and poor literacy, thus older adults tended to perceive the technologies as difficult to use [39].

Beyond the proposed research framework of the study, we aimed to use an integrated TAM and user satisfaction, similar to other studies [29,31,34]. However, due to the severe violation of normality observed in TAM 3 constructs, we cannot integrate the TAM in path analysis. Nonetheless, previous research has shown a significant influence of PEOU on user satisfaction, with the latter proposed to be a key predictor of BI [32-34]. Additionally, Chao [29] showed that perceived enjoyment, effort expectancy, and performance expectancy have a signiﬁcantly positive effect on satisfaction; thus, it would have been relevant to include these variables to predict satisfaction.

Regarding usability, Venkatesh et al [19,75] theorized that PEOU is affected by the objective usability of a specific system only after a direct experience with the system, where perceptions about the PEOU are determined solely by usability features, which in turn form the basis for acceptance or rejection. Moreover, if the system has higher objective usability, it means that system that is easy to use. Several studies suggested that usability is a determinant of PEOU [19,23,75].

Regarding study limitations, our sample is not representative of the entire older population because we used a convenience sample. Therefore, findings cannot be widely generalizable. Moreover, the population sample is more homogenous than the wider population on the common factors, possibly leading to attenuation in correlations or erroneous correlations among variables [76,77]. Although we have a minimum sample size adequate for the estimation method (>100 participants), the SEM is a large-sample technique [78]. Therefore, future studies should have a larger and more heterogeneous sample to obtain sufficiently accurate estimates, although our study had a larger sample size compared with previous ones [13,49-51]. A further limitation is that the user experience was assessed for a specific wearable activity tracker (Xiaomi Mi Band 2), and therefore, is not representative of the full range of devices currently available on the market. Moreover, the testing period was limited to 15 days. Short-term technology acceptance may not be indicative of long-term acceptance, as research indicates that use of activity trackers tend to drop after the first few weeks [1,49], with short timeframes also making it difficult to determine the impact of the novelty effect (defined as a person’s subjective “first responses to a technology, not the patterns of usage that will persist over time as the product ceases to be new” [79]). Moreover, research suggests that the declining novelty effect could be a reason for many activity tracker users discontinuing their use. Recently, Shin et al [80] explored the effect of novelty in the early stages (<3 months) of activity tracker adoption, as well as the motivation factors for sustained activity tracker use in the long term (>6 months). Findings reveal that the use beyond the novelty period is determined by intrinsic and extrinsic motivations. Finally, we selected the SUS for the usability evaluation because it is the most widely used questionnaire to measure perceived usability; however, this instrument does not comprise all of the concepts regarding usability. For instance, there are several different standards (eg, ISO-9241-11 [26], ISO/IEC 9126 [81]) and conceptual models to evaluate usability. Shackel [82] reported on the 4 important characteristics of usability, namely, effectiveness, learnability, flexibility, and attitude, and the Nielsen model (1993) [83] gave 5 subattributes of usability, namely, learnability, efficiency, memorability, errors, and satisfaction [84,85]. Therefore, there is a need for future studies evaluating key dimensions of usability.

### Conclusions

In conclusion, while there is a pressing need for studies to include other devices currently on the market and evaluate longer-term use, our study extended on the existing research providing valuable insight into the use of wearable activity trackers among older adults. First, a significant contribution of this work was to demonstrate the relevance of usability as an important factor influencing user satisfaction, which probably has an impact on technology acceptance and on the intention to use activity trackers. However, we were not able to predict BI in our structural model. Furthermore, our results emphasize the need to consider strategies to minimize the usability barriers to technology adoption in older adults. In addition, system designers should provide systems that address these concerns, and the researchers must ensure that selected systems adequately address the usability issues to be effectively implemented in clinical and research settings. Second, our study investigated the impact of user characteristics as moderating factors influencing the relationship between usability and user satisfaction and found that depression symptoms have a significant influence on older adults’ perception of using technology. However, other individual differences/personal user characteristics should be examined, and the identified moderating effects should be taken into consideration when implementing strategies trying to promote technology adoption. Finally, our results suggested that the Xiaomi Mi Band 2 is a suitable wearable activity tracker for older adults to use in real-life context.

## Appendix

Multimedia Appendix 1 Measurement items of TAM 3. TAM: Technology Acceptance Model.

Multimedia Appendix 2 Measurement items of USEQ. USEQ: User Satisfaction Evaluation Questionnaire.

Multimedia Appendix 3 Measurement items of SUS. SUS: System Usability Scale.

## Abbreviations

BI: behavioral intention
CANX: computer anxiety
CFA: confirmatory factor analysis
CFI: Comparative Fit Index
GDS: Geriatric Depression Scale
GFI: Goodness of Fit Index
MMSE: Mini-Mental State Examination
PA: physical activity
PEC: perceptions of external control
PEOU: perceived ease of use
PU: perceived usefulness
RMSEA: Root Mean Squared Error of Approximation
SEM: structural equation modeling
SRMR: Standardized Root Mean Squared Residual
SUS: System Usability Scale
TAM: Technology Acceptance Model
TLI: Tucker–Lewis Index
USEQ: User Satisfaction Evaluation Questionnaire
VIF: variance inflation factor
