# Supplementary material for: Usability, Acceptability, and Satisfaction of a Wearable Activity Tracker in Older Adults: Observational Study in a Real-Life Context in Northern Portugal
Source: J Med Internet Res. 2022 Jan 26;24(1):e26652. doi: 10.2196/26652 (PMC8829694; doi:10.2196/26652)
Supplement: Multimedia Appendix 1 [file jmir_v24i1e26652_app1.docx]

**Multimedia Appendix 1.** Measurement items of TAM 3. TAM: Technology Acceptance Model.

| **Construct** | **Code** | **Question** |
| --- | --- | --- |
| Perceived Ease of Use | PEOU 1 | My interaction with the system is clear and understandable. |
|  | PEOU 2 | Interacting with the system does not require a lot of my mental effort. |
|  | PEOU 3 | I ﬁnd the system to be easy to use. |
|  | PEOU 4 | I ﬁnd it easy to get the system to do what I want it to do. |
| Perceptions of External control | PEC1 | I have the resources necessary to use the system. |
|  | PEC2 | The system is not compatible with other systems I use. |
| Computer Anxiety | CANX1 | Computers do not scare me at all. |
|  | CANX2 | Working with a computer makes me nervous. |
|  | CANX3 | Computers make me feel uncomfortable. |
| Behavioral intention | BI | Assuming I had access to the system, I intend to use it.  Given that I had access to the system, I predict that I would use it. |
| Use | USE | On average, how much time do you spend on the system each day? |
