# Supplementary material for: Usability, Acceptability, and Satisfaction of a Wearable Activity Tracker in Older Adults: Observational Study in a Real-Life Context in Northern Portugal
Source: J Med Internet Res. 2022 Jan 26;24(1):e26652. doi: 10.2196/26652 (PMC8829694; doi:10.2196/26652)
Supplement: Multimedia Appendix 2 [file jmir_v24i1e26652_app2.docx]

**Multimedia Appendix 2.** Measurement items of USEQ. USEQ: User Satisfaction Evaluation Questionnaire.

| **Code** | **Question** |
| --- | --- |
| USEQ1 | Did you enjoy your experience with the system? |
| USEQ2 | Were you successful using the system? |
| USEQ3 | Were you able to control the system? |
| USEQ4 | Is the information provided by the system clear? |
| USEQ5 | Did you feel discomfort during your experience with the system? |
| USEQ6 | Do you think that this system will be helpful for your rehabilitation? |
