# Supplementary material for: Usability, Acceptability, and Satisfaction of a Wearable Activity Tracker in Older Adults: Observational Study in a Real-Life Context in Northern Portugal
Source: J Med Internet Res. 2022 Jan 26;24(1):e26652. doi: 10.2196/26652 (PMC8829694; doi:10.2196/26652)
Supplement: Multimedia Appendix 3 [file jmir_v24i1e26652_app3.docx]

**Multimedia Appendix 3.** Measurement items of SUS. SUS: System Usability Scale.

| **Code** | **Question** |
| --- | --- |
| SUS1 | I think that I would like to use this system frequently. |
| SUS2 | I found the system unnecessarily complex. |
| SUS3 | I thought the system was easy to use. |
| SUS4 | I think that I would need the support of a technical person to be able to use this system. |
| SUS5 | I found the various functions in this system were well integrated. |
| SUS6 | I thought there was too much inconsistency in this system. |
| SUS7 | I would imagine that most people would learn to use this system very quickly. |
| SUS8 | I found the system very cumbersome to use. |
| SUS9 | I felt very confident using the System. |
| SUS10 | I needed to learn a lot of things before I could get going with this system. |
